# Associations between Mode of HIV Testing and Consent, Confidentiality, and Referral: A Comparative Analysis in Four African Countries

**DOI:** 10.1371/journal.pmed.1001329

**Published:** 2012-10-23

**Authors:** Carla Makhlouf Obermeyer, Melissa Neuman, Alice Desclaux, Rhoda Wanyenze, Odette Ky-Zerbo, Peter Cherutich, Ireen Namakhoma, Anita Hardon

**Affiliations:** 1 Center for Research on Population and Health, American University of Beirut, Beirut, Lebanon; 2 Harvard School of Public Health, Boston, Massachusetts, United States of America; 3 Institut de Recherche pour le Développement, Dakar, Sénégal; 4 Makerere University School of Public Health, Kampala, Uganda; 5 Programme d'Appui au Monde Associatif et Communautaire, Ouagadougou, Burkina Faso; 6 National AIDS/STD Control Program, Ministry of Health, Nairobi, Kenya; 7 REACH Trust, Lilongwe, Malawi; 8 Anthropology of Care and Health, University of Amsterdam, Amsterdam, The Netherlands; Massachusetts General Hospital, United States of America

## Abstract

**Background:**

Recommendations about scaling up HIV testing and counseling highlight the need to provide key services and to protect clients' rights, but it is unclear to what extent different modes of testing differ in this respect. This paper examines whether practices regarding consent, confidentiality, and referral vary depending on whether testing is provided through voluntary counseling and testing (VCT) or provider-initiated testing.

**Methods and Findings:**

The MATCH (Multi-Country African Testing and Counseling for HIV) study was carried out in Burkina Faso, Kenya, Malawi, and Uganda. Surveys were conducted at selected facilities. We defined eight outcome measures related to pre- and post-test counseling, consent, confidentiality, satisfactory interactions with providers, and (for HIV-positive respondents) referral for care. These were compared across three types of facilities: integrated facilities, where testing is provided along with medical care; stand-alone VCT facilities; and prevention of mother-to-child transmission (PMTCT) facilities, where testing is part of PMTCT services. Tests of bivariate associations and modified Poisson regression were used to assess significance and estimate the unadjusted and adjusted associations between modes of testing and outcome measures. In total, 2,116 respondents tested in 2007 or later reported on their testing experience. High percentages of clients across countries and modes of testing reported receiving recommended services and being satisfied. In the unadjusted analyses, integrated testers were less likely to meet with a counselor before testing (83% compared with 95% of VCT testers; *p<*0.001), but those who had a pre-test meeting were more likely to have completed consent procedures (89% compared with 83% among VCT testers; *p<*0.001) and pre-test counseling (78% compared with 73% among VCT testers; *p = *0.015). Both integrated and PMTCT testers were more likely to receive complete post-test counseling than were VCT testers (59% among both PMTCT and integrated testers compared with 36% among VCT testers; *p<*0.001). Adjusted analyses by country show few significant differences by mode of testing: only lower satisfaction among integrated testers in Burkina Faso and Uganda, and lower frequency of referral among PMTCT testers in Malawi. Adjusted analyses of pooled data across countries show a higher likelihood of pre-test meeting for those testing at VCT facilities (adjusted prevalence ratio: 1.22, 95% CI: 1.07–1.38) and higher satisfaction for stand-alone VCT facilities (adjusted prevalence ratio: 1.15; 95% CI: 1.06–1.25), compared to integrated testing, but no other associations were statistically significant.

**Conclusions:**

Overall, in this study most respondents reported favorable outcomes for consent, confidentiality, and referral. Provider-initiated ways of delivering testing and counseling do not appear to be associated with less favorable outcomes for clients than traditional, client-initiated VCT, suggesting that testing can be scaled up through multiple modes without detriment to clients' rights.

*Please see later in the article for the Editors' Summary*

## Abstract 2

A study carried out by Carla Obermeyer and colleagues examines whether practices regarding consent, confidentiality, and referral vary depending on whether HIV testing is provided through voluntary counseling and testing or provider-initiated testing.

## Editors' Summary

**Background:**

In 2007, World Health Organization (WHO) and the Joint United Nations Programme on HIV/AIDS (UNAIDS) issued a joint guidance document on “provider-initiated” HIV testing and counseling. They noted that previous testing strategies that relied on “client-initiated” testing (also referred to as VCT, for voluntary counseling and testing) had failed to reach enough people, both in high-income and resource-constrained countries—in Africa, for example, at that time, just 12% of men and 10% of women had ever been tested. They argued that many opportunities to diagnose and counsel people that visit health facilities for other reasons are being missed, and that provider-initiated HIV testing and counseling can help expand access to HIV treatment, care, and support. They made it clear, however, that mandatory testing is not acceptable. All provider-initiated testing must therefore give individuals the option to not be tested. In addition, the guidelines stressed that all testing must continue to observe “the three Cs” (informed consent, counseling, and confidentiality) and be accompanied by an “enabling environment” including the availability of antiretroviral therapy, prevention and support services, and a supportive social, policy, and legal framework. A number of advocates have subsequently criticized the guidelines for failing to recognize that health-care services and staff in some countries do not always observe the three Cs. Critics have also questioned the appropriateness of the strategy for settings where antiretroviral therapy is not always available or where stigma and discrimination remain widespread.

**Why Was This Study Done?:**

To inform the debate surrounding scale-up of HIV testing in general and provider-initiated testing in particular with data on “real-life” testing, researchers have since carried out a number of studies. One of them, called MATCH (for Multi-Country African Testing and Counseling for HIV), was designed to allow systematic comparisons across African countries of different ways of HIV testing. Its goal was to investigate the uptake of testing, to analyze differences in the experience of testing across countries and modes of testing, and to use the results to devise better strategies to increase knowledge of HIV status and referral to care. MATCH used different means to collect information, including surveys and interviews. People from Burkina Faso, Kenya, Malawi, and Uganda participated. Some had undergone HIV testing, others had not. This study used a subset of the survey data collected for the MATCH study and asked whether there were systematic differences depending on the type of testing people had experienced.

**What Did the Researchers Do and Find?:**

The data the researchers used were from 2,116 people who had undergone testing in the two previous years at different facilities in the four countries. The different facilities were grouped into three “modes” of testing: VCT-only testing, integrated testing (which included hospitals and other medical facilities where provider-initiated and client-initiated testing were both available, along with other medical services), and prevention of mother-to-child transmission (PMTCT) testing at medical facilities offering services to pregnant women. Analyzing the survey responses, the researchers categorized them as related to eight different “outcomes”: pre-test meeting, pre-test counseling, consent, confidentiality, satisfaction with the person-to-person interactions, post-test meeting to receive results, post-test counseling, and referral to care.

They found that across countries and different facilities, the majority of participants reported having received most of the testing-related services. More than 90% reported having a pre-test meeting, and around 80% were satisfied with the personal interactions, with the consent process, and with confidentiality. About 50% of participants reported receiving all post-test services, and 71% of those who had tested positive for HIV reported appropriate referral to care.

When they looked for differences between different modes of testing, the researchers found that while they existed, they did not consistently favor one mode over another. Some outcomes scored higher in VCT facilities, some in PMTCT facilities, and some in integrated facilities.

**What Do These Findings Mean?:**

While there is room for improvement in HIV testing services (especially post-test services) across the countries and facilities included, the study did not reveal major problems with consent or confidentiality. The results also suggest that services at PMTCT and integrated facilities are not any worse than those at VCT-only sites. It seems therefore reasonable to continue expanding access to HIV testing and to include all facilities in the scale-up. That said, this is only one of a number of studies examining issues surrounding HIV testing, and decisions should be based on all available evidence. The results here are consistent with some of the other studies, but there are also reports that counseling might become neglected as testing is scaled up, and that offering testing routinely at every doctor's visit makes it seem mandatory even if there is the possibility to “opt out.” Other analyses of the MATCH study use in-depth interviews to understand in more detail the feelings, experiences, and attitudes of participants who have been tested as well as those who have not been tested. It will be important to see whether their results are consistent with the ones here, which are based on a survey of people who have been tested.

**Additional Information:**

Please access these websites via the online version of this summary at http://dx.doi.org/10.1371/journal.pmed.1001329.

- WHO has published a toolkit for scaling up HIV testing and counseling services in resource-limited settings, as well as the report Service Delivery Approaches to HIV Testing and Counselling (HSC): A Strategic HTC Programme Framework
- In response to reactions to the 2007 joint WHO/UNAIDS guidelines Guidance on Provider-Initiated HIV Testing and Counselling in Health Facilities, the UNAIDS Reference Group on HIV and Human Rights issued a Statement and Recommendations on Scaling up HIV Testing and Counselling
- The NAM/aidsmap website has a section on HIV testing policies and guidelines.

## Introduction

Since HIV tests became available in the mid-1980s, concerns about human rights have led to heated discussions among those promoting wider testing and those who fear the social consequences of positive diagnoses. In the early years of the epidemic, a degree of “exceptionalism” characterized policies around HIV, whereby public health measures that were considered normal for other diseases, including routine testing, were deemed unacceptable in the case of HIV, because of the potential for stigma and discrimination [1]. Proponents of wider testing maintained that traditional modes of voluntary counseling and testing (VCT) could not sufficiently expand access to treatment and prevention [2], but many human rights advocates were reluctant to endorse the routinization of testing or its expansion at health facilities. They argued that the potential benefits did not outweigh the risks, that only client-initiated VCT was acceptable, and that careful scrutiny of consent procedures and practices around confidentiality was necessary [3]–[6]. Despite the wider availability of treatment, there are continuing concerns about expanding testing if consent, confidentiality, and proper care cannot be guaranteed [1],[3].

Until recently, the evidence to inform debates around HIV testing was patchy, in part because of the lack of unified criteria to measure outcomes, particularly in sub-Saharan Africa. In addition, studies that examined the shift from client- to provider-initiated testing tended to focus on acceptance rates rather than on actual testing experience [7]. There have not been systematic examinations of differences in outcomes for VCT compared to provider-initiated testing and counseling (PITC).

In the past few years, growing interest in monitoring HIV testing has led to the formulation of guidelines to ensure that practices around informed consent, confidentiality, and care respect the rights of clients. In 2007, the World Health Organization published a guidance document on PITC, shortly after the US Centers for Disease Control and Prevention had developed their own guidelines, and in 2009, European countries formulated guidelines to define minimum standards of what should be provided everywhere [8]–[10] (see also the more recent summaries [11],[12]). Despite some variations, these international guidelines represent a useful basis to define a set of outcomes that are measurable across settings.

The MATCH (Multi-Country African Testing and Counseling for HIV) study was designed to conduct systematic comparisons across countries of different ways of providing HIV testing (referred to here as modes of testing). Its overall goals are to investigate the uptake of testing, to analyze differences in the experience of testing across countries and modes of testing, and to assess the implications of these results for policies designed to increase knowledge of HIV status and referral to care. The project formulated a number of specific aims regarding variations in services around testing, in motivations to test, and in the experience of testing and its consequences, as reported by clients and by providers. Different sorts of data were collected, ranging from large surveys of clients using precoded responses (used in this analysis) to qualitative data transcribed from semi-structured interviews with key informants (not used in this analysis). Analyses of MATCH data focus on particular subsets of the data, for example, those pertaining to attitudes towards testing, the extent of stigma, or the frequency of disclosure of HIV status. In this analysis, we focus on services related to testing and examine the extent to which consent, confidentiality, and referral to care vary across modes of testing, namely VCT and PITC.

MATCH was conducted in four countries—Burkina Faso, Kenya, Malawi, and Uganda—that exemplify different approaches to the provision of services. In all four countries, multiple government- and donor-supported initiatives have been implemented, and, increasingly, testing is conducted at health facilities. But testing practices reflect the countries' different histories with HIV testing and foreign aid, and there are differences in the pace of increase (somewhat slower in Burkina Faso), in the scale of the resources invested (larger in Kenya and Uganda), in the role of VCT (which still represents an important mode of provision in Burkina Faso and Uganda), and in the relative roles of governments and nongovernment organizations. All four countries face similar challenges with the expansion of testing: insufficient resources, infrastructure, and adequately trained staff; limited public awareness about the risks of HIV; and difficulties with referral and follow-up. But while fears of coercion and breaches of confidentiality are frequently invoked in the literature, it is unclear whether these differ depending on how testing is provided, and how clients feel regarding their experiences with different modes of testing.

This paper analyzes respondents' reports regarding consent, confidentiality, and referral, to assess whether these outcomes differ systematically by mode of testing. Specifically, we assess whether eight outcome measures related to the completeness and quality of services—including the adequacy of pre- and post-test counseling, clients' consent to test, confidentiality, satisfactory interactions with providers, and referral for those HIV positive—differ between provider-initiated and client-initiated modes of testing. The null hypothesis, that there are no significant differences, would suggest that inadequate outcomes are not more likely when testing is scaled up at health facilities.

## Methods

### Ethics Statement

The study was approved by the Ethics Review Committee of the World Health Organization and by an Institutional Review Board (IRB) in each of the four countries (Burkina Faso's Comité d'Ethique pour la Recherche en Santé of the Ministries of Health and Higher Education, Kenyatta National Hospital's IRB in Kenya, the National Health Sciences Research Committee of the Ministry of Health and Population in Malawi, and the IRB of Makerere University and the National Council for Science and Technology in Uganda). Informed consent was obtained from all respondents who were invited to participate in the study. In Burkina Faso and Uganda, consent was in writing for virtually all respondents, except for a few illiterate respondents, who provided a thumbprint; in Malawi and Kenya, a greater proportion of respondents gave oral consent, consistent with local practices and, in the case of Malawi, because of higher illiteracy. Because the proportion of oral consent was relatively high in Kenya (24%), special permission to use the data from those interviews was obtained from the IRB. No identifying information was collected as part of the study dataset.

### Study Design and Sample

The core component of the MATCH study is a client survey, conducted in 2008–2009 at health facilities. It was designed to represent users of health facilities in the capital city and one province in each country, and to include individuals who had tested and others who had not tested for HIV. In each country, the research team, which comprised individuals knowledgeable about HIV testing, drew up a list of health facilities or services within large facilities that provided HIV testing, and facilities or services known not to provide HIV testing. The list of facilities for testers included the major providers of testing services and different types of facilities, namely, stand-alone facilities for VCT and integrated facilities where testing was provided along with prenatal care, tuberculosis treatment, or general medical care. Non-testers were recruited at medical facilities or services where HIV testing was not provided. Testing status was ascertained for all respondents. Other components of the MATCH study, not incorporated into this analysis, include smaller surveys of clients tested at home or during campaigns, interviews with providers, and semi-structured interviews with selected clients and providers.

About 20 facilities or services within larger facilities were selected for inclusion in each country. Adult clients (aged 18 y and over) who were present at the selected facilities on the appointed days were invited to participate in the study. All respondents who agreed to discuss their experience with testing or not testing for HIV were included. Response rates are estimated at 80% in Burkina Faso and 90% in Malawi and Uganda; the figure for Kenya is considerably lower, at about one-half, reflecting the difficult political situation prevailing in 2008, which translated into poor security, reluctance to participate, and cancelling or aborting of interviews.

The instrument used in the client survey included closed- and open-ended questions about socio-demographic information; attitudes towards HIV testing and counseling; testing status; for those previously tested, a checklist on services received during pre- and post-test meetings and follow-up care; and interactions with providers and experiences before, during, and after testing, including disclosure and stigma. The interview was piloted in the four countries and revised in light of field experience to ensure that the questions were understood and the sequence of questions was adequate. Interviews lasted approximately 30 min.

A total of 3,659 respondents were interviewed at the selected facilities in the four countries. Because we were interested in recent testing experience across different modes of testing, this analysis focuses on respondents who reported testing at different sorts of facilities in the 2 y preceding the survey. We excluded non-testers (1,088); testers who reported testing before 2007 or who were missing information on test date or testing status (385); those missing data on place of test or whose place of test could not be categorized, for example, if they tested abroad (34); and those missing information on covariates (36). Thus, the analysis includes 2,116 respondents tested at different types of facilities in 2007 or later. Each outcome was analyzed using a complete case method. Denominators are not the same for all outcome measures, for example, respondents who did not meet with a counselor before their test were not eligible to answer questions about the quality of pre-test counseling. The final sample values are found in Table 1. The analysis is stratified by country, because differences in policy environments and in the implementation of HIV testing made it plausible that there would be different associations in the four countries between test modes and the outcome measures in which we were interested.

**Table 1:** Percent respondents reporting on outcome measures of HIV testing, by country and mode of testing.

Country	Outcome Measure	Integrated (Percent)	VCT (Percent)	PMTCT (Percent)	Total (*n*)	Total (Percent Yes)	χ2(2)	*p*-Value
**All countries**	Pre-test: meet with counselor	83.3	95.3	92.3	1,875	89	66.18	<0.001
	Pre-test index: all items	78.6	73.1	72.3	1,410	75.4	8.38	0.015
	Consent index: all items	89.7	83.4	82.9	1,611	86.1	16.51	<0.001^b^
	Post-test: received results	98.6	100	99	1,997	99.1	9.04	0.011
	Post-test index: all items	58.7	35.8	59	1,015	51.5	90.25	<0.001
	Confidentiality index: all items	81.6	77	77.6	1,451	79.2	5.34	0.069
	Referral index^a^: all items	73.7	66.2	63.3	393	70.6	4.52	0.105
	Satisfaction index: all items	80.2	79.9	83	1,647	80.7	1.78	0.411
**Burkina Faso**	Pre-test: meet with counselor	67.4	96.3	96.2	538	88.8	86.24	<0.001^b^
	Pre-test index: all items	45.7	62.4	40.2	476	54.4	18.43	<0.001^b^
	Consent index: all items	77.2	74.8	83.3	476	77.1	3.07	0.215
	Post-test: received results	95.6	100	100	537	98.9	17.72	<0.001^b^
	Post-test index: all items	7.8	15.1	14.2	525	13.1	4.28	0.118
	Confidentiality index: all items	75	77.4	64.4	463	74.1	6.56	0.038
	Referral index^a^: all items	63.8	50.8	83.3	114	57.9	3.52	0.172
	Satisfaction index: all items	62.1	77.0	77.1	516	73.4	10.79	0.005^b^
**Kenya**	Pre-test: meet with counselor	97	97	92.4	376	96	3.39	0.183
	Pre-test index: all items	82.8	75	82.2	361	82	1.18	0.555
	Consent index: all items	95.3	93.8	90.4	361	94.2	2.50	0.286
	Post-test: received results	99.2	100	100	376	92.4	0.86	0.649
	Post-test index: all items	79.1	68.8	82.1	363	78.8	2.44	0.296
	Confidentiality index: all items	78.8	72.7	74	356	77.2	1.17	0.557
	Referral index^a^: all items	81.4	77.8	87.5	130	81.5	0.27	0.872
	Satisfaction index: all items	83.1	87.5	88.6	366	84.7	1.60	0.448
**Malawi**	Pre-test: meet with counselor	87	97.7	91.4	529	89	5.49	0.064
	Pre-test index: all items	92.1	90.5	89	471	91.1	1.06	0.588
	Consent index: all items	94.4	88.1	88.2	471	92.1	5.76	0.056
	Post-test: received results	99.4	100	99.3	524	93.3	0.30	0.860
	Post-test index: all items	71.2	53.5	75.2	517	70.8	7.53	0.023^b^
	Confidentiality index: all items	94.9	85.7	92	462	93.3	5.43	0.066
	Referral index^a^: all items	66.2	57.1	56.3	175	64	1.26	0.533
	Satisfaction index: all items	95.3	90.7	93.5	520	94.4	1.84	0.398
**Uganda**	Pre-test: meet with counselor	72.7	93.9	89.4	664	85.1	51.97	<0.001^b^
	Pre-test index: all items	67.2	80.8	77.4	562	75.8	11.62	0.003^b^
	Consent index: all items	80.6	89.8	67.5	563	83.5	25.02	<0.001^b^
	Post-test: received results	98.2	100	96.6	578	98.8	7.23	0.027^b^
	Post-test index: all items	45.9	52.1	67.4	565	52	11.48	0.003^b^
	Confidentiality index: all items	67.0	75.8	75.3	552	72.8	4.61	0.100
	Referral index^a^: all items	86.0	78.4	57.1	138	79	5.52	0.063
	Satisfaction index: all items	65.0	80.4	68.5	640	73	17.29	<0.001^b^

a Includes only HIV-positive respondents.

b Remains significantly less than 0.05 after correction for false discovery rate.

This analysis is designed to assess the extent to which respondents' experiences with testing are consistent with prevalent standards for the quality and completeness of the services to be provided along with HIV testing. We draw on data collected in response to questions and checklists regarding respondents' testing experience, including the information, advice, and referral that were provided during pre- and post-test counseling, interaction with providers, and the extent to which clients' rights were protected by practices regarding consent and confidentiality. We assess whether there are differences in these responses for clients tested at different types of testing facilities, adjusting for respondents' socioeconomic characteristics and for the clustering of responses in interview facilities.

### Outcome Measures

Primary outcomes in this study included eight measures of the quality and completeness of the testing process for each respondent. Unified guidelines for testing were not available for the four countries at the time the study was designed, but we drew on the documents issued by the World Health Organization and the US Centers for Disease Control and Prevention [8],[9] to define the elements that were generally thought to ensure adequate care and to protect individuals' rights during and after HIV testing.

The “3 Cs” framework refers to consent, confidentiality, and counseling as essential elements in HIV testing [13]. In view of discussions regarding how much pre-test counseling is necessary, and the need to tailor post-test counseling to the test results [8],[9],[14], we examined the adequacy of pre- and post-test counseling separately. Thus, pre- and post-test counseling, consent, and confidentiality are the first four outcomes. We used clients' reports on their interactions with providers—whether they thought the information provided was sufficient, whether they were well treated, and whether they found the meeting useful—to create a fifth outcome measure that we referred to as satisfaction. The sixth outcome measure, referral, captures the extent to which testing was linked to further care for HIV-positive respondents. In addition, because pre-test measures depend on having had a pre-test meeting and post-test measures on receiving results, we also analyzed the likelihood of these two additional outcome measures where appropriate, for a total of eight outcome measures.

We used respondents' responses to specific questions in the interview to create an index for each of these outcome measures. Because we wanted to assess whether each required element was present, we gave equal importance to the items that defined each index, and coded the index as complete if all items were present, and as incomplete if any was absent. The outcome measures and the individual items that make up each index are summarized in Box 1.

**Box 1. Outcome Measures Regarding Pre- and Post-Test Counseling and Referral**

Indices of Pre- and Post-Test Counseling and Referral*

**1. Pre-test counseling index**

Whether the provider:

- Explained how HIV is transmitted
- Explained the meaning of positive and negative results
- Gave time to ask questions

**2. Post-test counseling index**

Whether the provider:

- Explained the meaning of the results
- Gave advice on prevention
- Advised discussing test results with significant others
- Advised partner referral
- Gave time to ask questions

**3. Consent index**

Whether the provider:

- Asked if respondent agreed to test
- Explained that respondent had a choice to accept or refuse

**4. Confidentiality index**

- Whether provider explained that results would not be shared
- Whether respondent believed results had been protected

**5. Satisfaction with post-test counseling index**

Whether respondent:

- Thought s/he received sufficient information
- Thought s/he was well treated
- Thought the meeting was useful

**6. Referral index (only for HIV-positive individuals)**

Whether respondent:

- Was referred for care during post-test counseling
- Was ever prescribed medications

**Additional Outcome Measures**

**7. Pre-test meeting**

Whether the respondent had a pre-test meeting

**8. Post-test results received**

Whether the respondent received results in post-test meeting

*Based on the questions as phrased in the questionnaire.

### Independent Variables

The key independent variable used is the mode of testing. The different types of testing facilities were grouped into three modes of testing: (1) integrated testing, which included hospitals and medical facilities where provider-initiated and client-initiated testing were both provided, along with medical services; (2) stand-alone VCT; (3) prevention of mother-to-child transmission (PMTCT) testing, which included antenatal clinics and other facilities offering care to pregnant women; additionally, all women who reported testing in integrated testing facilities because of pregnancy were coded as PMTCT testers.

Other covariates that may have influenced clients' experiences of testing and were adjusted for in the analyses included age, education, and wealth. Age was self-reported and measured in years. Education was specified as no schooling, incomplete or completed primary education, or some secondary education. We developed a country-specific wealth index using principal components analysis. The wealth index methodology is based on the Filmer-Pritchett wealth score methodology developed for Demographic and Health Surveys [15], which incorporates dichotomous variables into a wealth score using factor analysis. This method has been widely used in international health surveys and has been established as a reliable proxy for household wealth. Respondents' household assets (television, electric or gas stove, telephone, land, animals) and amenities (tap water, flush toilet, electricity) were converted into *z*-scores, and factor loadings for a single wealth factor were calculated. Values of the indicator variables were multiplied by the factor loadings to obtain a wealth score for each respondent. Within each country, respondents were grouped into quartiles by wealth score.

### Analysis Plan

We estimated the unadjusted and adjusted effects of mode of testing on the eight outcome measures. The effect of mode of testing on referral to subsequent care is estimated for HIV-positive respondents only. Unadjusted associations were tested using chi-squared tests, and Fisher's exact tests of significance were also used in analyses with cell sizes of five or fewer respondents. In many cases, more than 10% of respondents in each group had tested for HIV, so odds ratios estimated using logistic regression analysis would not provide a reasonable estimate of the relative prevalence of testing. Consequently, to estimate adjusted associations between mode of testing and index scores we used a modified Poisson regression analysis with robust standard errors [16]. We report 95% confidence intervals for all parameters and exact *p*-values from Wald tests of significance for all tests where *p*≥0.001. Given the number of comparisons, our analyses also adjusted for false discovery rate using the Benjamini, Krieger, and Yekutieli step-up adjustment method [17], as implemented using the smileplot add-on to Stata 10 [18] and an unadjusted *p*-value of 0.05.

All regression analyses estimated the effect of mode of testing, and we compared VCT and PMTCT to integrated testing as the reference category. Regression analyses were adjusted for country, using a fixed effect (fixed effect parameters and constants not shown), and standard errors were adjusted for clustering of responses at the interview facility, using a generalized estimating equation. All analyses were completed in Stata, release SE 10.1 [19]. This report adheres to STROBE guidelines (see Text S1).

## Results

The distribution of respondents by testing mode differs across countries. More respondents tested at stand-alone VCT sites than at integrated sites in Burkina Faso (55% [293/537] and 26% [138/537], respectively) and Uganda (47% [315/664] and 38% [255/664]), while more tested at integrated sites than at stand-alone VCT sites in Malawi (65% [348/533] and 8% [43/533], respectively) and Kenya (70% [268/382] and 9% [34/382]). PMTCT testers ranged from 14% (94/664) in Uganda to 27% (142/533) in Malawi. (Detailed frequencies of respondents' age, sex, educational attainment, and wealth score quartiles, by country and mode of testing, are presented in Table S1.)

Table 1 presents the frequencies of respondents' reports on the eight outcome measures across all countries and for each country.

Across all countries, respondents in integrated testing were less likely to meet with a counselor before their HIV test (83% compared with 95% of VCT testers; *p<*0.001), but those who did meet with a counselor were more likely to have completed consent procedures (89% compared with 83% among VCT testers; *p<*0.001) and pre-test counseling (78% compared with 73% among VCT testers; *p = *0.015). Both integrated and PMTCT testers were more likely to receive complete post-test counseling than were VCT testers (59% among both PMTCT and integrated testers compared with 36% among VCT testers; *p*<0.001). Differences among the three groups in proportion referred to care, proportion satisfied with their post-test counseling, and proportion believing that confidentiality had been ensured were not significant.

Examining the unadjusted associations between testing mode and outcome measures by country reveals some notable differences, as shown in Table 1. In Burkina Faso, integrated testers were significantly less likely to meet with a counselor before their test (67% compared with 96% among VCT testers; *p*<0.001), to have a complete pre-test index (46% compared with 62% among VCT testers; *p*<0.001), and to be satisfied with post-test counseling (62% compared with 77% of VCT testers and 77% of PMTCT testers). In Uganda, integrated testers were less likely to meet with a counselor (73% compared with 94% among VCT testers; *p<*0.001), to have complete pre-test counseling (67% compared with 81% among VCT testers and 77% among PMTCT testers; *p = *0.003), to have complete post-test counseling (46% compared with 52% among VCT testers and 67% among PMTCT testers; *p = *0.003), and to be satisfied with post-test counseling (65% compared with 80% among VCT testers and 69% among PMTCT testers; *p<*0.001). In Malawi, VCT testers were less likely to receive full post-test counseling than PMTCT or integrated testers (54% of VCT testers compared with 71% of integrated testers and 75% of PMTCT testers; *p = *0.023). In Kenya there were no significant differences across testing modes.

Table 2 presents the effects of mode of testing on the eight outcome measures, after adjustment for respondent age, sex, educational attainment, and wealth. In Burkina Faso, satisfaction with post-test counseling remained significantly higher among VCT testers than among integrated testers; the adjusted prevalence ratio (APR) comparing VCT testers to integrated testers was 1.28 (95% CI: 1.10–1.48). In Uganda, respondents testing in stand-alone VCT sites were significantly more likely to meet with a counselor (APR: 1.25; 95% CI: 1.04–1.51), to have completed confidentiality procedures (APR: 1.13; 95% CI: 1.04–1.23), and to be satisfied with their post-test counseling experience (APR: 1.23; 95% CI: 1.10–1.37), while HIV-positive PMTCT testers were significantly less likely to receive referrals for additional care (APR: 0.71, 95% CI: 0.57–0.89). In Malawi, respondents testing in stand-alone VCT sites were significantly more likely to meet with a counselor before testing (APR: 1.11; 95% CI: 1.05–1.18).

**Table 2:** Prevalence ratios for outcome measures, by country and mode of testing.

Outcome Measure	Adjusted Prevalence Ratio (95% CI)				
	All Countries	Burkina Faso	Kenya	Malawi	Uganda
**Pre-test meeting**					
Integrated testing	1.00	1.00	1.00	1.00	1.00
Stand-alone VCT	1.22^a^	1.45	0.90	1.11^a^	1.25
	(1.07–1.38)	(0.94–2.22)	(0.72–1.12)	(1.05–1.18)	(1.04–1.51)
PMTCT	1.09	1.41	0.97	1.03	1.17
	(1.00–1.20)	(0.93–2.13)	(0.85–1.10)	(0.96–1.09)	(0.93–1.47)
**Pre-test index complete**					
Integrated testing	1.00	1.00	1.00	1.00	1.00
Stand-alone VCT	1.15	1.34	0.90	0.97	1.18
	(0.99–1.33)	(0.86–2.10)	(0.72–1.12)	(0.85–1.10)	(0.97–1.44)
PMTCT	0.98	0.88	0.97	0.96	1.15
	(0.88–1.09)	(0.59–1.31)	(0.85–1.10)	(0.87–1.06)	(0.91–1.45)
**Consent index complete**					
Integrated testing	1.00	1.00	1.00	1.00	1.00
Stand-alone VCT	1.03	1.01	1.00	0.94	1.1
	(0.95–1.12)	(0.82–1.24)	(0.93–1.08)	(0.88–1.01)	(0.96–1.27)
PMTCT	0.95	1.08	0.94	0.93	0.86
	(0.89–1.02)	(0.89–1.31)	(0.82–1.09)	(0.87–1.00)	(0.66–1.11)
**Post-test results received**					
Integrated testing	1.00	1.00	1.00	1.00	1.00
Stand-alone VCT	1.02	1.05	1.00	1.01	1.02
	(1.00–1.04)	(0.99–1.10)	(1.00–1.01)	(1.00–1.03)	(1.00–1.04)
PMTCT	1.00	1.04	1.00	0.99	0.98
	(0.99–1.01)	(0.99–1.08)	(0.99–1.00)	(0.98–1.01)	(0.95–1.02)
**Post-test index complete**					
Integrated testing	1.00	1.00	1.00	1.00	1.00
Stand-alone VCT	1.00	1.93	0.91	0.78	1.05
	(0.83–1.20)	(0.90–4.10)	(0.69–1.21)	(0.59–1.03)	(0.86–1.28)
PMTCT	1.09	1.87	1.04	1.00	1.40
	(0.94–1.27)	(0.83–4.24)	(0.91–1.20)	(0.85–1.17)	(1.01–1.94)
**Confidentiality index complete**					
Integrated testing	1.00	1.00	1.00	1.00	1.00
Stand-alone VCT	1.04	1.02	0.99	0.91	1.13^a^
	(0.97–1.11)	(0.90–1.15)	(0.82–1.18)	(0.79–1.05)	(1.04–1.23)
PMTCT	0.97	0.88	0.94	0.97	1.14
	(0.90–1.06)	(0.76–1.02)	(0.71–1.26)	(0.91–1.03)	(0.96–1.36)
**Referral index complete** b					
Integrated testing	1.00	1.00	1.00	1.00	1.00
Stand-alone VCT	0.87	0.76	0.90	0.84	0.91
	(0.74–1.02)	(0.50–1.16)	(0.78–1.04)	(0.57–1.22)	(0.78–1.07)
PMTCT	0.89	1.56	0.88	0.89	0.71^a^
	(0.73–1.08)	(0.87–2.78)	(0.64–1.20)	(0.62–1.26)	(0.57–0.89)
**Satisfaction index complete**					
Integrated testing	1.00	1.00	1.00	1.00	1.00
Stand-alone VCT	1.15^a^	1.28^a^	1.02	0.97	1.23^a^
	(1.06–1.25)	(1.10–1.48)	(0.87–1.21)	(0.88–1.06)	(1.10–1.37)
PMTCT	1.05	1.19	1.06	0.97	1.07
	(0.98–1.13)	(1.03–1.38)	(0.92–1.22)	(0.92–1.03)	(0.80–1.43)

All models adjusted for respondent sex, age (in years), educational attainment, and wealth index, and adjusted for clustered sampling using a generalized estimating equation. All countries model also adjusted for country using fixed effect.

a *p*-Value remains significantly less than 0.05 after correction for false discovery rate.

b Includes only HIV-positive respondents.

Analyses of the pooled data across countries show a higher likelihood of a pre-test meeting for VCT (APR: 1.22; 95% CI: 1.07–1.38) compared to integrated testing, and higher satisfaction for VCT compared to integrated testing (APR: 1.15; 95% CI: 1.06–1.25). Other differences were not statistically significant.

## Discussion

First, a major result of this analysis is that across all countries and modes of testing, high percentages of clients reported having received the services around testing that are considered important in most guidelines. As shown in Table 1, across the four countries, 89% reported having a pre-test meeting, and more than 75% reported a complete pre-test index. Consent, confidentiality, and overall satisfaction indices were high, at 86%, 79%, and 81%, respectively. Provision of post-test services was generally lower: though 99% received their results, just over half reported a complete post-test index, and 71% of HIV-positive respondents reported a complete referral index. This suggests that there is still a good deal of room for improvement to provide advice and referral after HIV testing, but that services around testing do not appear to be associated with major breaches of autonomy or privacy. When we compared reports by HIV-positive and HIV-negative respondents, we found them to be similar, and where they differed, HIV-positive respondents reported more favorably on the services received, which does not support the notion that HIV-positive individuals receive worse services due to stigma.

A second major result of this analysis is that there were some significant associations between outcome measures and modes of testing, but differences across modes of testing were not consistent with the notion that one mode of testing performs better than the others. As shown in Tables 1 and 2, unadjusted analyses indicate somewhat more favorable reports at integrated testing sites on a few outcome measures, while analyses adjusting for covariates and clustering of facilities show slightly higher likelihood of better outcomes for VCT. In general, the results do not support the notion that testing at integrated health facilities functions less adequately than at stand-alone VCT sites.

Taken together, these results suggest that the rights of clients, particularly regarding informed consent and confidentiality, can be protected even as testing is scaled up at health facilities. Some studies have suggested that consent and confidentiality may be jeopardized by inadequate facilities and procedures, poor counseling, or the power differential between providers and patients [20]–[27], while other studies have documented high acceptability and improved referral, with no reduction in the utilization of health services when testing is routinized [7],[28]–[30]. The results of this analysis are consistent with studies showing that routine testing is compatible with ethical practices [31],[32], and this should provide some reassurance about the possibility of scaling up HIV testing through PITC without compromising individual protection.

The association of VCT with more positive outcome measures than integrated or PMTCT testing in Uganda—specifically pre- and post-test counseling, receiving results, and satisfaction—can be attributed to the role of nongovernment organizations, which mobilized early on, when VCT was the only mode of provision, to encourage testing, increase awareness, and fight stigma and discrimination. The less favorable outcomes for pre- and post-test counseling in Burkina Faso may be attributable to the more recent initiation of PITC in the country, which had, for a long time, relied on VCT provided via freestanding facilities and national campaigns. PITC facilities are comparatively less resourced in Burkina Faso, and the guidelines in place at the time of the survey did not recommend all of the post-test items that we included in our indices. Instead, the guidelines in Burkina Faso referred to explaining the meaning of results, providing advice on prevention, and answering questions. When measured against more demanding standards, testing services fell short. These results underscore the need to clarify and harmonize guidelines on testing and counseling and to support their implementation.

The data were collected in 2008–2009, at an important time for the scale-up of HIV testing programs. In the countries where the study was conducted, as well as globally, 2006–2007 was a turning point for the increased availability of treatment, the formulation of guidelines, and the implementation of programs to increase knowledge of HIV status. The results reflect the scale-up of these efforts and are likely indicative of subsequent trends.

The generally favorable outcomes that are documented in this study suggest that it is possible to expand client- and provider-initiated testing while protecting patients' rights. This should not be surprising. The standards defined by guidelines on testing, such as informing clients, obtaining consent, and providing information, are not especially exacting, and can be achieved in most settings. Other studies, such as in the United States [33],[34] and in Egypt [35], have reported similarly high levels of service provision ad satisfaction.

It is possible that the high values we found in reported measures of satisfaction result in part from a contrast with clients' general experience with other health services, which tends to be suboptimal, so that, by comparison, the quality HIV testing services appears to be satisfactory. It is also possible that because respondents were interviewed at health facilities, they may have felt that they should not appear ungrateful for the services they receive, and hence may have given more favorable answers. In order to assess the extent of such social desirability bias, we conducted a sensitivity analysis comparing outcomes among respondents who tested at facilities where interviews were conducted and those who reported testing at facilities not included in the MATCH project. We found that outcomes among respondents tested at MATCH facilities tend to be similar to, or less positive than, those who did not test at MATCH facilities, suggesting that the facility environment did not substantially influence respondents' reports on the care they received.

Other studies in Kenya and Malawi have found that counseling may be neglected when testing is scaled up, and that clients may perceive testing as compulsory [36],[37]. Qualitative data such as were used in these studies are important complements to surveys. They provide insights into potential gaps in the functioning of services, highlighting research questions regarding counseling and consent for further investigation and suggesting areas for improvement. Analyses are underway on the texts transcribed from responses to open-ended questions in the MATCH survey, and from the semi-structured interviews carried out in parallel with the survey. The texts provide a complementary way to assess clients' views, and the analyses should yield more detailed insights into the reasons for clients' positive or negative evaluations of their testing experiences.

There are limitations to this study, including the fact that the outcome measures may not account for important social, cultural, and health service variations. Our measures of referral and medication use do not include specific details, and hence provide only a rough measure of referral. In addition, our analysis distinguishes modes of testing as discussed in the literature, and compares VCT and PITC (including integrated and PMTCT testing), but the reality of services on the ground is complex, and the boundaries among types of testing facilities are not always strict. It is also possible that the lack of significant associations between testing mode and outcomes at the level of individual countries reflects the smaller numbers of respondents in some of the categories of testing, and that larger samples may have been able to detect more associations.

A further limitation comes from using a sample of convenience, based on systematic rather than random selection, and hence not necessarily representative. This strategy was deemed to be appropriate, since the goal was to compare testing experiences across modes of testing. MATCH respondents are more urban, educated, and wealthy than respondents to national surveys—except in Malawi, where they are similar [38]–[41]. This is not surprising, since users of health facilities tend to have higher socioeconomic status than the general population. Thus, there are reasons to believe that these results are not gross overestimates and that they reflect the reality of testing in the selected areas.

A strength of this study is that it used the same outcome measures and methodology across sites. Hence, results can be compared across countries to address policy questions about modes of testing. While there are considerable differences, both within and among countries, in the structural factors that define health services and accessibility and the meanings attached to testing, the motivations behind it, and its consequences, there is a degree of consistency in the results that likely reflects the state of services around testing in these different settings.

### Conclusion

Despite variations within and across countries in the outcome measures for pre- and post-test counseling, consent, confidentiality, referral, and satisfaction, respondents' reports indicate that the frequencies of positive responses on these measures are generally high, suggesting that overall, people testing for HIV do not appear to experience adverse outcomes such as coercion or breaches of confidentiality, and that the majority of people who are tested are satisfied with the services they receive.

The fact that we found few consistent associations between mode of testing and outcome measures is also encouraging. Provider-initiated modes of testing (integrated medical testing and PMTCT testing) were not more likely to be associated with less favorable outcome measures than VCT. These results should provide some reassurance that the efforts to provide HIV testing in different countries through different approaches can help improve knowledge of HIV status and referral to appropriate care.

## Supporting Information

Table S1 **Frequencies of age, sex, educational attainment, and wealth quartiles by country and mode of testing.**
(DOCX)

Text S1 **STROBE statement.**
(DOC)

## Abbreviations

APR: adjusted prevalence ratio
IRB: Institutional Review Board
PITC: provider-initiated testing and counseling
PMTCT: prevention of mother-to-child transmission
VCT: voluntary counseling and testing
