# Supplementary material for: Associations between Mode of HIV Testing and Consent, Confidentiality, and Referral: A Comparative Analysis in Four African Countries
Source: PLoS Med. 2012 Oct 23;9(10):e1001329. doi: 10.1371/journal.pmed.1001329 (PMC3479110; doi:10.1371/journal.pmed.1001329)
Supplement: Table S1 — Frequencies of age, sex, educational attainment, and wealth quartiles by country and mode of testing. (DOCX) [file pmed.1001329.s001.docx]

**Supplemental Table S1**

**Frequencies (percent)* of age, sex, educational attainment, and assets quartiles by country and mode of testing**

|  |  | | |  | | | |  | | | |  | | | |  | | | | |  | | | | | |  | | | | | |  | | | | |  | | | | | | |  | | | |  | | | | | |
| --- | --- | --- | --- | --- | --- | --- | --- | --- | --- | --- | --- | --- | --- | --- | --- | --- | --- | --- | --- | --- | --- | --- | --- | --- | --- | --- | --- | --- | --- | --- | --- | --- | --- | --- | --- | --- | --- | --- | --- | --- | --- | --- | --- | --- | --- | --- | --- | --- | --- | --- | --- | --- | --- | --- |
|  | | **Burkina Faso** | | | | | | | | | **Kenya** | | | | | | | | | **Malawi** | | | | | | | | | | | | | **Uganda** | | | | | | | | | | | | | | **Total** | | | |  | |  |  |
|  | | **Integ-rated** | **VCT** | | | **PMTCT** | | | **Total** | | **Integ-rated** | | **VCT** | **PMTCT** | | | | **Total** | | **Integ-rated** | | | **VCT** | | | **PMTCT** | | | | **Total** | | | **Integ-rated** | | | **VCT** | | | | **PMTCT** | | | **Total** | | | |  | | | |  | |  |  |
| **Age (yrs) mean** | | 33.3 | 30.8 | | 25.4 | | 30.4 | | | | 31.3 | | 28 | | 28.4 | | 30.4 | | | 33.1 | | 29 | | 26.5 | | | | 31 | | | | | 33.3 | 30.1 | | | 25.1 | | | | 30.6 | | | | | | 30.6 | | | | | | |  |
| SD | | 9.2 | 9.1 | | 4.9 | | 8.9 | | | | 7.6 | | 7.6 | | 6.2 | | 7.4 | | | 11.3 | | 11.2 | | 6.6 | | | | 10.7 | | | | | 12 | 8.7 | | | 5.4 | | | | 10.1 | | | | | | 9.5 | | | | | | |  |
|  | |  |  | |  | |  | | |  |  | |  | |  | |  | |  |  | |  | |  | | | |  | | |  | |  |  | | |  | | | |  | | |  | | |  |  | | | | | |  |
|  | | **%** | **%** | | **%** | | **No.** | | | **%** | **%** | | **%** | | **%** | | **No.** | | **%** | **%** | | **%** | | **%** | | | | **No.** | | | **%** | | **%** | **%** | | | **%** | | | | **No.** | | | **%** | | | **No.** | **%** | | | | | |  |
| **Age (10-yr cats)** | |  |  | |  | |  | | |  |  | |  | |  | |  | |  |  | |  | |  | | | |  | | |  | |  |  | | |  | | | |  | | |  | | |  |  | | | | | |  |
| 0-24 | | 12.3 | 29.7 | | 43.4 | | 150 | | | 27.9 | 17.5 | | 47.1 | | 29.1 | | 86 | | 22.5 | 25.0 | | 34.9 | | 46.5 | | | | 168 | | | 31.5 | | 23.5 | 31.4 | | | 51.1 | | | | 207 | | | 31.2 | | | 611 | 28.9 | | | | | |  |
| 25-34 | | 47.8 | 38.9 | | 53.8 | | 237 | | | 44.1 | 56.1 | | 29.4 | | 58.2 | | 207 | | 54.2 | 36.5 | | 51.2 | | 43 | | | | 210 | | | 39.4 | | 40 | 40 | | | 42.6 | | | | 268 | | | 40.4 | | | 922 | 43.6 | | | | | |  |
| 35-44 | | 27.5 | 22.5 | | 2.8 | | 107 | | | 19.9 | 17.8 | | 17.6 | | 10.1 | | 62 | | 16.2 | 23.9 | | 7 | | 8.5 | | | | 98 | | | 18.4 | | 19.2 | 20.3 | | | 6.4 | | | | 119 | | | 17.9 | | | 386 | 18.2 | | | | | |  |
| 45+ | | 12.3 | 8.9 | | 0 | | 43 | | | 8 | 8.6 | | 5.9 | | 2.5 | | 27 | | 7.1 | 14.7 | | 7 | | 2.1 | | | | 57 | | | 10.7 | | 17.3 | 8.3 | | | 0 | | | | 70 | | | 10.5 | | | 197 | 9.3 | | | | | |  |
|  | |  |  | |  | |  | | |  |  | |  | |  | |  | |  |  | |  | |  | | | |  | | |  | |  |  | | |  | | | |  | | |  | | |  |  | | | | | |  |
| **Sex of the respondent** | |  |  | |  | |  | | |  |  | |  | |  | |  | |  |  | |  | |  | | | |  | | |  | |  |  | | |  | | | |  | | |  | | |  |  | | | | | |  |
| Female | | 55.8 | 58.4 | | 100 | | 354 | | | 65.9 | 41.3 | | 55.9 | | 100 | | 209 | | 54.7 | 62.9 | | 25.6 | | 100 | | | | 372 | | | 69.8 | | 52.9 | 52.1 | | | 100 | | | | 393 | | | 59.2 | | | 1328 | 62.8 | | | | | |  |
| Male | | 44.2 | 41.6 | | 0 | | 183 | | | 34.1 | 58.7 | | 44.1 | | 0 | | 173 | | 45.3 | 37.1 | | 74.4 | | 0 | | | | 161 | | | 30.2 | | 47.1 | 47.9 | | | 0 | | | | 271 | | | 40.8 | | | 788 | 37.2 | | | | | |  |
|  | |  |  | |  | |  | | |  |  | |  | |  | |  | |  |  | |  | |  | | | |  | | |  | |  |  | | |  | | | |  | | |  | | |  |  | | | | | |  |
| **Education (3 cats)** | |  |  | |  | |  | | |  |  | |  | |  | |  | |  |  | |  | |  | | | |  | | |  | |  |  | | |  | | | |  | | |  | | |  |  | | | | | |  |
| No formal education | | 37 | 20.5 | | 40.6 | | 154 | | | 28.7 | 0.4 | | 2.9 | | 0 | | 2 | | 0.5 | 19.5 | | 9.3 | | 16.2 | | | | 95 | | | 17.8 | | 7.1 | 7 | | | 8.5 | | | | 48 | | | 7.2 | | | 299 | 14.1 | | | | | |  |
| Primary incomplete/complete | | 31.2 | 19.8 | | 34.9 | | 138 | | | 25.7 | 30.5 | | 23.5 | | 41.8 | | 123 | | 32.2 | 56.3 | | 46.5 | | 66.9 | | | | 311 | | | 58.3 | | 47.1 | 25.1 | | | 46.8 | | | | 243 | | | 36.6 | | | 815 | 38.5 | | | | | |  |
| Secondary or more | | 31.9 | 59.7 | | 24.5 | | 245 | | | 45.6 | 69.1 | | 73.5 | | 58.2 | | 257 | | 67.3 | 24.1 | | 44.2 | | 16.9 | | | | 127 | | | 23.8 | | 45.9 | 67.9 | | | 44.7 | | | | 373 | | | 56.2 | | | 1002 | 47.4 | | | | | |  |
|  | |  |  | |  | |  | | |  |  | |  | |  | |  | |  |  | |  | |  | | | |  | | |  | |  |  | | |  | | | |  | | |  | | |  |  | | | | | |  |
| **Assets index (quartiles, country-specific)** | | | | | | |  | | |  |  | |  | |  | |  | |  |  | |  | |  | | | |  | | |  | |  |  | | |  | | | |  | | |  | | |  |  | | | | | |  |
| Lowest | | 17.4 | 17.1 | | 30.2 | | 106 | | | 19.7 | 24.5 | | 23.5 | | 19 | | 89 | | 23.3 | 20.1 | | 16.3 | | 26.1 | | | | 114 | | | 21.4 | | 21.2 | 15.6 | | | 16 | | | | 118 | | | 17.8 | | | 426 | 20.1 | | | | | |  |
| Second | | 34.1 | 25.6 | | 31.1 | | 155 | | | 28.9 | 21.6 | | 26.5 | | 24.1 | | 86 | | 22.5 | 25.3 | | 16.3 | | 28.9 | | | | 136 | | | 25.5 | | 41.2 | 21 | | | 26.6 | | | | 196 | | | 29.5 | | | 574 | 27.1 | | | | | |  |
| Third | | 27.5 | 25.3 | | 26.4 | | 140 | | | 26.1 | 30.9 | | 20.6 | | 29.1 | | 113 | | 29.6 | 25.3 | | 23.3 | | 26.1 | | | | 135 | | | 25.3 | | 17.6 | 23.2 | | | 29.8 | | | | 146 | | | 22 | | | 502 | 23.7 | | | | | |  |
| Highest | | 21 | 32.1 | | 12.3 | | 136 | | | 25.3 | 23 | | 29.4 | | 27.8 | | 94 | | 24.6 | 29.3 | | 44.2 | | 19 | | | | 148 | | | 27.8 | | 20 | 40.3 | | | 27.7 | | | | 204 | | | 30.7 | | | 614 | 29.0 | | | | | |  |
|  | |  |  | |  | |  | | |  |  | |  | |  | |  | |  |  | |  | |  | | | |  | | |  | |  |  | | |  | | | |  | | |  | | |  |  | | | | | |  |
| **Total (n)** | | **138** | **293** | | **106** | | **537** | | | **100** | **269** | | **34** | | **79** | | **382** | | **100** | **348** | | **43** | | **142** | | | | **533** | | | **100** | | **255** | **315** | | | **94** | | | | **664** | | | **100** | | | **2116** | **100** | | | | | |  |
|  | |  |  | |  | |  | | |  |  | |  | |  | |  | |  |  | |  | | |  | | | |  | | |  |  | |  | | | |  | | |  | | | |  |  | | |  | |  |  |  |

*All figures in the table are percentages, except for age and standard deviation of mean age, which are in years, and the Total columns which include number (No.) and percent.
